# Collagen epineurial substitute-assisted neurorrhaphy is superior to conventional end-to-end neurorrhaphy in a rat model of peripheral nerve injury

**DOI:** 10.1016/j.jot.2026.101197

**Published:** 2026-09-21

**Authors:** Yongquan Wang, Zoran Pletikosa, Peilin Chen, Jiayue Li, Ziming Chen, Cameron Bright, Sharon Chu, Alex O'Beirne, David Brogan, Brendan F. Kennedy, Harry Hoyen, Haotian Li, Daohui Li, Clair Lee, Lingqiang Chen, Rui Ruan, Minghao Zheng

**Affiliations:** a Centre for Orthopedics Research, The University of Western Australia, Nedlands, Western Australia, 6009, Australia; b Department of Orthopedics, The First Affiliated Hospital of Kunming Medical University, Kunming, Yunnan, 650031, China; c Perron Institute for Neurological and Translational Science, Nedlands, Western Australia, 6009, Australia; d Department of Electrical, Electronic & Computer Engineering, School of Engineering, University of Western Australia, Perth, Western Australia, 6009, Australia; e BRITElab, Harry Perkins Institute of Medical Research, QEII Medical Centre, Centre for Medical Research, University of Western Australia, Nedlands, Perth, Western Australia, 6009, Australia; f Medical School, Western Sydney University, Penrith, New South Wales, 2751, Australia; g Institute of Physics, Faculty of Physics, Astronomy and Informatics, Nicolaus Copernicus University, Toruń, 87-100, Poland; h Plastic and Reconstructive Surgery, Joondalup Health Campus, Joondalup, Western Australia, 6027, Australia; i Western Orthopaedic Clinic, Nedlands, Western Australia, 6009, Australia; j Department of Orthopaedic Surgery, Medical School, Washington University, St Louis, MO, 63110, USA; k Department of Orthopedics, The Cleveland FES Centre, Case Western Reserve University, Cleveland, OH, 44106, USA; l School of Medicine, Monash University, Clayton, Victoria, 3800, Australia

**Keywords:** Peripheral nerve injuries, Nerve regeneration, Mechanical properties, Neurorrhaphy, Collagen nerve conduit, Microenvironment

## Abstract

**Background:**

Peripheral nerve injuries (PNIs) are common and may result in profound and persistent functional deficits. Although end-to-end neurorrhaphy remains the gold standard for nerve repair, clinical outcomes are frequently suboptimal. This study aimed to validate a novel surgical method using a collagen epineurial substitute (CES) to minimize the number of sutures and reduce nerve tension at the coaptation site.

**Methods:**

Finite element analysis (FEA) was performed to simulate stress distribution and guide suture configuration. Sprague–Dawley (SD) rats underwent sciatic nerve transection and were allocated into six groups: control; end-to-end neurorrhaphy using 1, 3, or 6 epineurial sutures; or epineurial substitute-assisted repair with one or no coaptation-site sutures. Mechanical stability was validated by tensile testing, while the Sciatic Functional Index (SFI) and gastrocnemius muscle weight were used as screening assessments to evaluate functional recovery. Histological outcomes, including vascularization and cellular alignment at the transection site, were also assessed. A further comparison between two representative groups, based on the equivalent mechanical stability was performed using behavioural tests for sensory recovery and electrophysiological testing for motor recovery to precisely track functional outcomes, together with Osmium staining for myelin and Silver staining for nerve fibres, to further investigate nerve tissue regeneration associated with microenvironmental changes.

**Results:**

To test if CES-assisted repair can stabilize the mechanical strength and reduce nerve tension at the coaptation site, we first used FEA and demonstrated that, under physiological tension (2%), CES-assisted nerve coaptation redistributed mechanical stress along the covering of nerve segment. In contrast, conventional neurorrhaphy showed the concentrated tension of nerve at the coaptation. These computational findings were further supported by biomechanical testing in the rat model. The failure load of constructs repaired with three coaptation-site sutures was not significantly different from that of epineurial substitute-assisted constructs without coaptation-site sutures (p = 0.0635) and was significantly lower than that of CES-assisted constructs incorporating a single coaptation-site suture (p = 0.0022). This optimised mechanical environment offered by CES-assisted neurorrhaphy led to a 63–82% increase in parallel direction ratios relative to suture-only repairs, thereby promoting superior cellular alignment at the nerve transection site, enhancing epineurial and intraneural vascularization and reducing suture-related inflammation. In contrast, excessive suturing impaired early regenerative responses, whereas the epineurial substitute approach facilitated a tension-free microenvironment conducive to axonal regeneration and tissue integration. Repair of transected nerves with epineurial substitute demonstrated accelerated sensory functional recovery, reflected by improved SFI, earlier responses to thermal and mechanical stimuli, significantly higher maximum evoked compound muscle action potential (CMAP) of the gastrocnemius muscle (P = 0.0034), preservation of distal nerve calibre, and improved fascicular organization compared with suture-only repairs.

**Conclusion:**

Repair of transected nerves using an epineurial substitute can mechanically stabilize the coaptation site and reduce the nerve tension compared with conventional end-to-end neurorrhaphy, thereby preserving alignment of fascicular architecture, promoting early pro-regenerative vascularization, minimizing suture-related trauma and enhancing functional recovery.

**The translational potential of this article:**

The integration of computational modelling and biomechanical validation in this study reveals a new way of repairing nerve using CES-assisted neurorrhaphy with less suture. The approach transfer point-load fixation in the end-to-end neurorrhaphy to distributed-load stabilization with less nerve tension at the coaptation site. While the study showed a translational potential for changing current clinical practice in peripheral nerve repair, further validation in large animal models and clinically relevant settings are required before application in routine surgical practice.

## Graphical abstract

**Figure:**
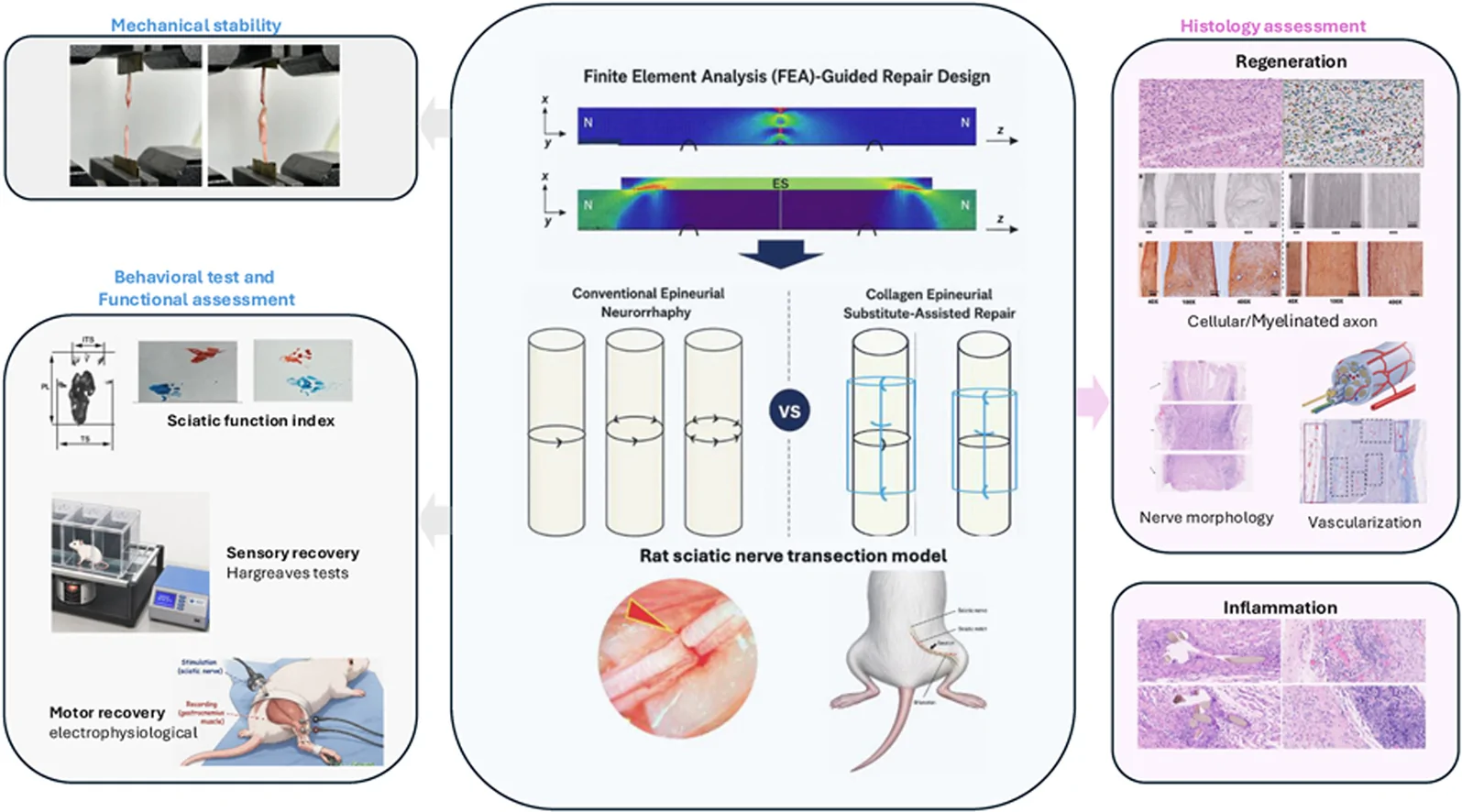

## 1 Introduction

Peripheral nerve injuries (PNIs) are a major global health issue, accounting for approximately 2.3% of traumatic injuries [1]. PNI can result in severe functional deficits, including persistent loss of motor, sensory, and autonomic function [2], significantly impairing patients’ quality of life [3], imposing a substantial global healthcare burden.

The goal of surgical treatment for PNIs is to promptly restore neural continuity without tension, thus direct regenerating axons into the distal stump, and minimizing fiber loss at the repair interface [4,5]. Currently, end-to-end neurorrhaphy is the most widely adopted surgical technique [6] and remains the gold standard for the repair of transected peripheral nerves [7,8]. Use of sutures is fundamental to a tension-free repair, but end-to-end neurorrhaphy with sutures often results in incomplete functional recovery, with substantial interpatient variability. In mixed motor-sensory nerve injuries, conventional neurorrhaphy is associated with suboptimal clinical outcomes [9], with only 40-60% of patients achieving satisfactory recovery [10].

It has been well documented that sutures elicit foreign-body inflammatory reactions at the coaptation site, leading to granuloma formation and subsequent scar tissue that disturbs the tension of nerve fibers, impedes axonal regeneration and accelerates nerve degeneration [11,12]. In addition, microsurgical suturing is technically demanding and time-consuming. Excessive manipulation or inaccurate epineurial suturing may disrupt fascicular alignment or cause secondary sharp injury to nerve and neuroma formation, ultimately resulting in poor functional recovery and neuropathic pain [[13], [14], [15]].

We hypothesized that directing sutures away from the coaptation interface can reduce suture associated mechanical stress and thus create a tension-free and pro-regenerative microenvironment that enhances peripheral nerve recovery. The aim of this study was to investigate whether a use of a collagen epineurial substitute (CES) can move the away from the coaptation site in nerve repair and still maintain coaptation of the nerve. We first simulated dynamic mechanical stress distribution at the nerve coaptation site using finite element analysis (FEA). This provided a theoretical foundation for optimizing suture configurations. We then developed a surgical repair technique to eliminate or reduce sutures using a CES (Remplir™). Unlike other collagen conduits or wrap devices used for nerve repair, Remplir™ has similar structural characteristics to epineurium and is able to directly engage with nerve to form the additional epineurial structure [16]. To test if sutures can be positioned away from the coaptation site without compromising the mechanical strength of the repair, we used a rat sciatic nerve transection model to compare conventional neurorrhaphy with different coaptation techniques using CES-assisted nerve repair. Using constructs of similar mechanical stability, we evaluated tissue- and cellular-level responses related to inflammation and nerve repair, and functional sensory and motor recovery.

## 2 Materials and methods

### 2.1 Materials

All surgical procedures were performed using a stereomicroscope (Aosvi SZM-41 L, Aosvi, China). Epineurial neurorrhaphy was carried out with non-absorbable 10-0 monofilament nylon sutures, which were used for all microsurgical suturing steps throughout the study.

For wrap-assisted nerve repair, a non-crosslinked type I collagen epineurial substitute of porcine origin (Remplir™, Orthocell Ltd, Australia) was applied. The collagen epineurial substitute (CES) is engineered as a selectively permeable construct that conforms closely to nerve tissue. The epineurial substitutes were prepared in accordance with the manufacturer's instructions for use. According to the instructions for use, the nerve wrap is biocompatible according to the requirements of ISO 10993 and has sufficient tensile strength to hold sutures.

### 2.2 Finite element analysis (FEA)

We hypothesized that the epineurial substitute may redistribute mechanical stress and provide necessary structural support to the coaptation site following end-to-end repair of a transected sciatic nerve. To test this hypothesis, two-dimensional axisymmetric (z-axis) static finite element models of suture repair and epineurial substitute-assisted repair were developed using ANSYS Mechanical (ANSYS Inc., Canonsburg, PA, USA; v2021R1). For illustrative purposes, the geometry of the nerve segment was modelled as a cylindrical structure with a radius of 1 mm and a finite length of 5 mm, assuming near incompressibility (Poisson's ratio ν = 0.49) and a linear elastic modulus (Young's modulus) of 14.6 MPa, consistent with previously reported mechanical characteristics of human peripheral nerve tissue [17]. The collagen epineurial substitute was modelled as a cylindrical sheet with a thickness of 300 μm and a length of 7 mm, extending past the coaptation region between the transected nerve ends. A linear elastic material model was adopted, with a Young's modulus of 17.9 MPa, which was characterised through independent tensile testing of Remplir™. All geometries were discretised using quadrilateral finite elements with a uniform mesh size of 50 μm. All models were subjected to an axial displacement corresponding to a nominal bulk strain of 2%, which is within the reported physiological loading range of peripheral nerves [[18], [19], [20]]. The resulting stress fields were then computed by solving the governing equations of linear elasticity under static conditions.

To compare stress distributions between suture repair and CES-assisted repair, four finite element models with different contact and constraint configurations were constructed. Conventional neurorrhaphy was represented by single-suture and two-suture repair models. In model 1 (single-suture), the two nerve segments were joined by a single bonded contact region at the coaptation site. In model 2 (two-suture), two bonded contact regions were defined at the nerve-nerve interface, separated by 250 μm along the axial direction. In contrast, the CES assisted repair groups were modelled both with and without coaptation-site suturing. In model 3 (CES without coaptation suture), the nerve segments were enclosed by the CES without direct bonding at the nerve-nerve interface. Low-friction contact (coefficient of friction = 0.05) was assigned between the nerve ends to allow relative sliding, while bonded contact was defined at the distal ends of the CES-nerve interfaces to represent fixation of the epineurial substitute. In model 4 (CES with single coaptation suture), in addition to the epineurial substitute configuration described above, a bonded contact region was introduced at the two nerve segments at the coaptation site, consistent to the first single-suture repair case.

For all models, von Mises stress was calculated to visualise and compare non-uniform stress distributions in the vicinity of the coaptation site.

### 2.3 FEA guided experimental animal study

The design of the animal study is summarized in Fig. S1. In all experimental groups (Group A-E), the left sciatic nerve was sharply transected at a site approximately 1 cm proximal to the nerve bifurcation. In Groups A, B, and C, end-to-end epineurial neurorrhaphy was performed using different suture numbers to generate graded suture burdens. Nerves were repaired using different techniques: a single epineurial suture in Group A, three sutures at 120° intervals in Group B, and six sutures at 60° intervals in Group C (Fig. 1e–g). In Group D, following nerve transection, the nerve stumps were aligned on CES, which had been pre-cut to 10 mm × 8 mm and immersed in saline immediately prior to use. The nerve stumps were approximated with a single epineurial suture. The CES was wrapped around the two nerve ends and closed with two anchor sutures placed 1 mm from the proximal and distal ends of the wrap, entering and exiting the epineurium at a distance of 0.5-1 mm proximal and distal to the edge of the nerve wrap. An additional fixation suture was placed at the midpoint of the CES to tighten the construct and close the tubular configuration longitudinally; this suture passed exclusively through the CES without contacting the nerve or epineurium. In Group E, the CES wrapped around the nerve and anchor sutures was performed at the distal and proximal sites similar to Group D, without placement of an epineurial suture at the transection site (Fig. 1h and i). In the sham group (Group F), the sciatic nerve was exposed using the same surgical procedure but received no further intervention (Fig. 1 j).

**Fig. 1:**
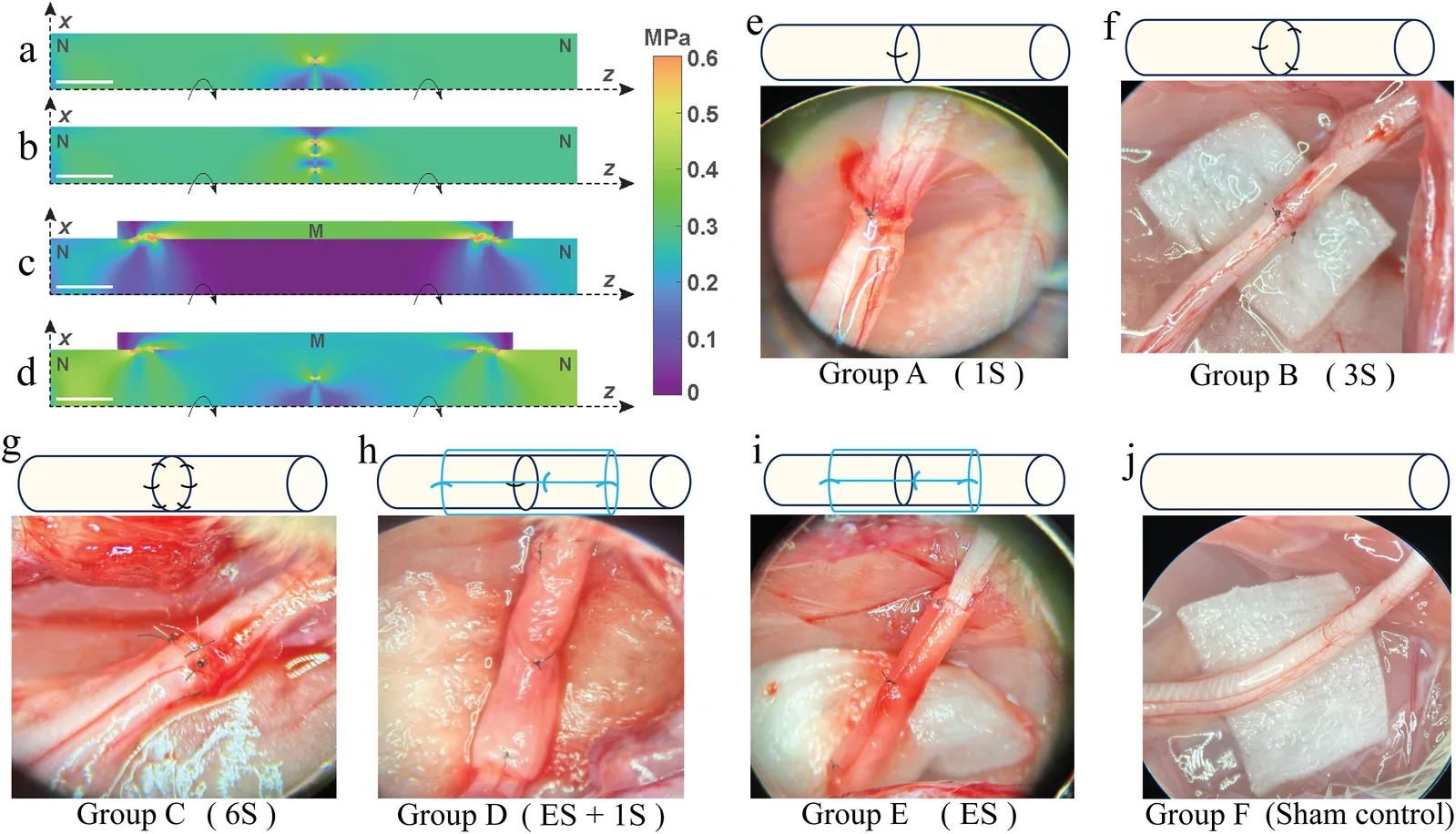
**FEA models and experimental procedures for nerve repair. (a-d)** Simulated von Mises stress distributions at the coaptation site for: **(a)** single-suture, **(b)** two-suture, **(c)** sutureless epineurial substitute-assisted, and **(d)** single-suture epineurial substitute-assisted repairs. (N: Nerve; M: Epineurial Substitute Membrane. Scale bars represent 1 mm). **(e-j)** Schematic illustrations (upper) and representative intraoperative images (lower) of surgical groups. **(e-g)** End-to-end epineurial neurorrhaphy with increasing numbers of sutures. **(h, i)** Collagen epineurial substitute-assisted repair. **(j)** Sham control. (S: suture).

For longitudinal functional and histological evaluation of suture configuration using CES in nerve repair, a total of 97 female Sprague–Dawley rats (250–300 g) were randomly allocated to the five experimental groups (Groups A–E; *n* = 17 per group), with 12 rats assigned to the sham-operated control group (Group F). Immediately after surgery, five rats from each experimental group (Groups A–E) were randomly selected and euthanized for early tissue harvesting for mechanical testing. The remaining animals (*n* = 12 per group) were used for longitudinal functional and histological evaluation according to the predefined experimental timeline (4 and 12 weeks). Animal experiments were conducted in accordance with protocols approved by the Animal Care and Ethics Committee of Kunming Medical University (Approval #KMMU20231515).

For behavioural and electrophysiological assessments of suture configuration using CES, two representative experimental groups were further investigated: neurorrhaphy with 3 sutures to secure the coaptation site (Group B2), which closely reflects current clinical practice and equivalent repair stability, and epineurial substitute-assisted repair with no sutures at the transection site but with Remplir wrapped around the nerve as described above (Group E2), which represents the maximal modification to the conventional neurorrhaphy technique. A sham control group (Group F2), in which the sciatic nerve was exposed but received no transection, was also included. Fifteen female rats (Sprague–Dawley rats 250–300 g) were randomly allocated into each of these three groups. Behavioural and electrophysiological assessments were performed on 15 rats (*n* = 5 per group). Rats were euthanized 4, 8, and 17 weeks post-surgery (*n* = 9 per group, n = 3 at each time point) and the transected sciatic nerve was harvested to perform histological evaluation of nerve regeneration. The study was approved by the Western Sydney University Animal Care and Ethics Committee (ACEC: A10622).

### 2.4 Surgical procedure

All surgical procedures were performed under general anesthesia using an inhalation anesthesia system. Anesthesia was induced with 4% isoflurane in 100% oxygen (1 L/min) and maintained with 2.0-2.5% isoflurane (0.6 L/min) delivered via a nose cone. Adequate anesthetic depth was confirmed prior to surgery. Animals were placed in the prone position, and the surgical field was shaved, disinfected with povidone-iodine, and prepared under sterile conditions.

A longitudinal skin incision (approximately 3 cm) was performed using a surgical blade, initiated 1 cm inferior to the greater trochanter and extending distally toward the tibiofemoral joint. Following the lateral aspect of the femur at a 0.5 cm offset, the biceps femoris and gluteal muscles were separated via blunt dissection under an operating microscope. The sciatic nerve was then meticulously exposed and mobilized from the adjacent connective tissue to ensure minimal trauma. In all experimental groups (Groups A-E,B2,E2), the sciatic nerve was sharply transected approximately 1 cm proximal to its trifurcation using microsurgical scissors. The specific repair for each group was then performed as described above. In all cases, meticulous alignment of the nerve stumps was ensured to achieve proper fascicular apposition without tension, using 10-0 nonabsorbable monofilament nylon sutures. Following nerve repair, the surgical field was irrigated with sterile saline. Muscle and skin layers were closed separately using 4-0 absorbable sutures, and the skin was disinfected with povidone-iodine. The sham group (Group F and F2) underwent an identical surgical procedure, with the sole exception of the nerve intervention. Animals were monitored postoperatively until full recovery from anesthesia. After recovery, rats were housed in separate plastic cages with polypropylene solid floored base and stainless-steel high-top lid with plentiful bedding material and free access to food and water. Postoperative animal husbandry conditions were strictly standardized across all groups. Animals were maintained under identical housing conditions with controlled nutrition, activity space, and unrestricted cage mobility to ensure comparable spontaneous limb use.

While all subjects survived the perioperative period, one animal in Group A died unexpectedly at week 10 and was excluded from subsequent analyses. No other mortalities or surgical complications were observed across the remaining groups.

Surgical procedures were performed by accredited orthopaedic surgeons with subspecialty expertise in microsurgery and spine surgery, both possessing extensive clinical experience and advanced microsurgical proficiency. To minimize operator-dependent variability, all procedures were conducted using a standardized surgical protocol, including uniform anesthesia, exposure technique, neurorrhaphy configuration, and suture materials.

At each time point of study, animals were sacrificed with a lethal dose of sodium pentobarbital (100 mg/kg) by intraperitoneal injection.

### 2.5 Mechanical testing

Rats (n = 5) from each experimental group (Groups A-E) were randomly selected for mechanical testing and were euthanized immediately following surgery. The repaired sciatic nerve segment was harvested from the operated side and immediately immersed in 0.9% isotonic saline for transport to the biomechanics laboratory. Nerve diameter was measured using a digital caliper and recorded.

Each nerve was mounted between two sandpaper tabs (1.5 × 1.0 cm) using cyanoacrylate adhesive to minimize grip slippage. The gauge length was standardized to 10 mm, and at least 5 mm of nerve tissue was embedded within each adhesive interface. A 300-g compressive load was applied to the sandpaper-nerve interfaces to ensure uniform bonding, and specimens were allowed to cure for 30 min [13]. Throughout preparation, tissues were continuously kept moist with isotonic saline to prevent dehydration [13,18].

Mechanical testing was conducted according to ASTM D882. Briefly, a universal testing machine (Instron® 34TM-10) was used, equipped with a calibrated load cell at a controlled room temperature (26 °C). Specimens were aligned vertically and loaded along the long axis. After applying a 0.03 N preload to remove slack and standardize initial tension, displacement was zeroed. Samples were elongated at a constant crosshead speed of 0.17 mm/s until failure. Load and displacement were recorded at 100 Hz and exported as CSV files for subsequent analysis [19,20].

Failure load was defined as the peak load recorded immediately before construct failure/rupture (Fig. 2c–h). Stress and strain were derived from load-displacement data using the measured nerve diameter and the initial gauge length. Elasticity modulus was calculated as the slope of the linear region of the stress-strain curve.

**Fig. 2:**
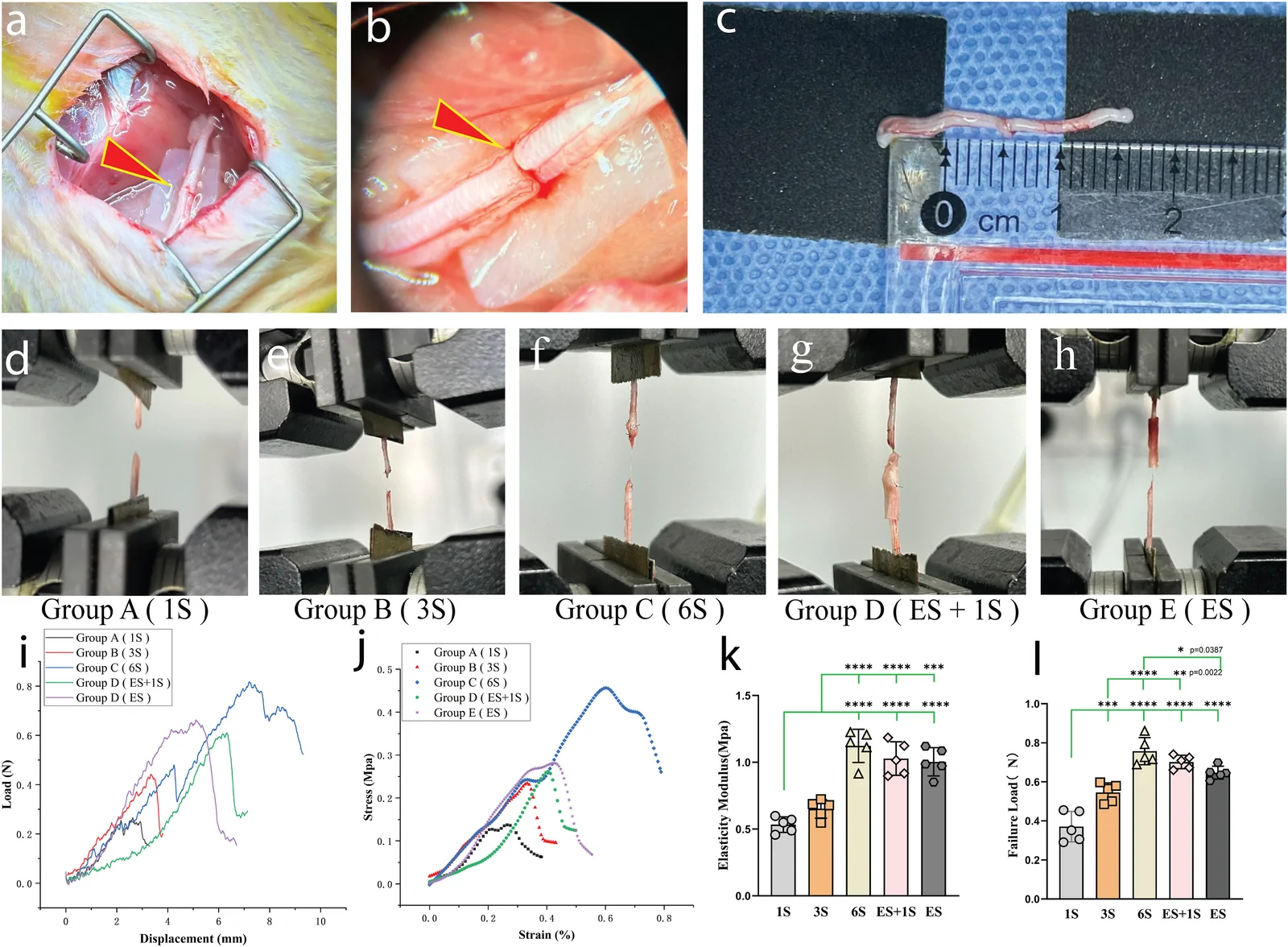
**Intraoperative alignment and mechanical failure characteristics across different nerve repair strategies. (a, b)** Representative gross **(a)** and microscopic **(b)** images showing the alignment of transected nerve stumps on the collagen epineurial substitute (Remplir™), highlighting stable apposition prior to suturing. **(c)** Experimental setup for mechanical tensile testing, showing the initial nerve length between sandpaper-lined grips. **(d-h)** Representative images capturing the failure modes of the five experimental groups (Groups A-E) at the onset of mechanical failure. **(i, j)** Representative load-displacement **(i)** and stress-strain **(j)** curves obtained during tensile testing. **(k, l)** Quantitative comparison of elastic modulus **(k)** (one-way ANOVA (F _(4, 20)_ = 33.11, p < 0.001; Brown-Forsythe test, p = 0.6432) followed by Tukey's post-hoc test. Exact adjusted p-values are reported. * = p < 0.05, ** = p < 0.01, *** = p < 0.001, **** = p < 0.0001, ns = not significant) and failure load **(l)** (one-way ANOVA (F _(4, 20)_ = 37.15, p < 0.001; Brown-Forsythe test, p = 0.4166) followed by Tukey's post-hoc test. Exact adjusted p-values are reported. * = p < 0.05, ** = p < 0.01, *** = p < 0.001, **** = p < 0.0001, ns = not significant) among the five groups (n = 5 per group).

### 2.6 Histology and quantitative image analysis

Sciatic nerve specimens were processed for longitudinal sectioning encompassing the repair region and adjacent normal nerve proximal and distal to the coaptation site. Sections were stained with hematoxylin and eosin (H&E) and Masson's trichrome, or Osmium and Modified Palmgren's silver staining using standard protocols. Images were acquired under a digital microscope.

For longitudinal functional and histological evaluation, quantitative histological analysis was performed on six nerves per group at each time point (4 and 12 weeks post-surgery). For each nerve, three longitudinal sections were randomly selected. One predefined region of interest (ROI) with a uniform area and shape was analysed per section, and measurements were averaged for quantitative analysis. For behavioural and electrophysiological assessments, five animals per group were assessed for the function for up to 17 weeks post-surgery.

Morphometric remodelling for evaluation of distal nerve calibre preservation following transection and repair, was assessed on H&E-stained sections by calculating the proximal-to-distal transverse diameter ratio across the repair region. Measurements were obtained from longitudinal sections at the first locations immediately proximal and distal to the repair interface where nerve calibre and fascicular morphology had returned to a normal appearance, thereby capturing changes across the transition from the repaired segment to structurally intact nerve. Higher ratios indicate a greater reduction in distal calibre relative to the proximal segment.

Cell alignment at the transection site was quantified using CellProfiler software. The nucleus of each cell in the predefined ROI was identified, and the orientation and angle of the nuclear longitudinal axis relative to the nerve's morphological alignment was calculated automatically by the software. Cells with orientation angles between 0° and 10° or 170°-180° were classified as parallel to the nerve axis. The parallel direction ratio for each group was calculated as the number of parallel-aligned cells divided by the total number of cells within the predefined ROI.

Vascularization was evaluated on Masson's trichrome-stained sections using ImageJ, The epineurial and intraepineurial revascularization at the transection site were assessed by quantifying vessel density (n/mm^2^) and vessel area fraction (%) within the predefined ROI, similar to methods described in previous studies [21,22].

### 2.7 Functional recovery assessment

Functional recovery assessments were conducted using both sciatic functional index for the short-term study and sensory function test for a longer term study. As the primary longitudinal outcome measure to assess functional recovery following nerve repair, the Sciatic Functional Index (SFI) (n = 5 per group at each time point) and gastrocnemius muscle wet weight (n = 6 per group at each time point) were investigated. These measurements reflected integrated gait performance and overall hindlimb locomotor function for up to 12 weeks. We also performed behavioural tests and electrophysiological testing for up to 17 weeks to precisely track on two representative groups of sutures versus CES assisted repair to evaluate motor reinnervation, muscle preservation, and neuromuscular recovery. At the study endpoint, gastrocnemius muscle wet weight (n = 5 per group) and compound muscle action potentials (CMAPs) (n = 5 per group) were measured.

#### 2.7.1 Walking track analysis and sciatic functional index

Functional recovery following sciatic nerve repair was evaluated using walking track analysis and the Sciatic Functional Index (SFI) [23] at 2, 4, 8, and 12 weeks postoperatively. Rats were allowed to walk voluntarily through a linear enclosed runway lined with paper for footprint collection, following prior habituation and training. To distinguish bilateral footprints, the plantar surfaces of the operated and contralateral hind paws were coated with different water-based pigments. Rats performed multiple runs at each time point, and footprint sequences with clear, uninterrupted steps were selected for analysis. Footprint acquisition and measurements were performed by an investigator blinded to group allocation.

SFI values were calculated based on standard parameters of print length, toe spread, and intermediary toe spread derived from the operated and contralateral hind limbs. For each group at each time point, five animals (n = 5) were analysed.

#### 2.7.2 Behavioural tests for sensory recovery

Two behavioural tests (withdrawal threshold tests) were performed on three occasions at 3-day intervals prior to surgery to obtain baseline values. Following surgery, testing continued once weekly for 17 weeks. On each testing occasion, animals were allowed to acclimatize to the testing chamber for 10 min before assessment commenced.

Mechanical sensitivity was assessed using a dynamic plantar Von Frey aesthesiometer (Ugo Basile, Italy). Stimuli were applied to the plantar surface of both hind paws while avoiding the saphenous nerve territory to minimize false-positive responses [24]. Rats were placed on a wire mesh platform, and a 0.4 mm probe delivered increasing force (10 g/s) until paw withdrawal occurred or the maximal force limit of 50 g was reached. Withdrawal latency was recorded.

Thermal sensitivity was assessed using an infrared plantar algesimetry Hargreaves apparatus (Ugo Basile, Italy; intensity setting 20). Rats were placed on a transparent plexiglass platform, and infrared stimuli were applied to the same plantar testing site. Withdrawal latency was recorded, with a 20-s cut-off applied to prevent tissue injury [25].

#### 2.7.3 Gastrocnemius muscle weight

Denervation-related muscle atrophy was quantified by calculating the gastrocnemius muscle wet weight ratio at 4, 8, 12 and 17 weeks postoperatively. After euthanasia, bilateral gastrocnemius muscles were harvested, cleared of adherent connective tissue, and weighed immediately on an analytical scale. The wet weight ratio was calculated as the ratio of the operated-side gastrocnemius wet weight to the contralateral side. At least five animals per group at each time point were analysed.

#### 2.7.4 Electrophysiological test for motor recovery

Motor recovery was assessed by electromyographic measurement of the maximum evoked compound muscle action potential (CMAP) of the gastrocnemius muscle following electrical stimulation of the sciatic nerve [26]. Rats were anesthetized and the sciatic nerve was exposed as described previously. A pair of silver/silver-chloride stimulating electrodes was placed proximal to the repair site on operated nerves, while in sham controls, electrodes were positioned 1 cm proximal to the nerve bifurcation. A recording electrode was placed on the exposed gastrocnemius muscle at a standardized location across all groups and both sides. Electrical stimulation was delivered using square-wave pulses (0.2 ms duration, 1 Hz frequency) beginning at 10 μA and progressively increased until no further increase in muscle response exhibited. EMG signals were recorded using a Power1401 analogue-to-digital converter and Spike2 software (Cambridge Electronic Design, UK), and the maximum CMAP amplitude (mV) was quantified.

### 2.8 Statistical analysis

All statistical analyses were performed using GraphPad Prism (GraphPad Software, San Diego, CA, USA). Data are presented as mean ± standard deviation (SD). All tests were two-tailed, and p < 0.05 was considered statistically significant. For single-time-point outcomes across groups (biomechanical parameters, H&E-derived proximal-to-distal diameter ratio, CellProfiler-derived parallel direction ratio, Masson-derived vessel density and vessel area fraction, and gastrocnemius wet weight ratio at each endpoint), data normality and homogeneity of variance were assessed using the Shapiro–Wilk test and the Brown-Forsythe test, respectively. Since the assumption of variance homogeneity was satisfied across all key datasets (p > 0.05), ordinary one-way ANOVA followed by Tukey's multiple comparisons test was performed. Statistical significance was defined as adjusted p < 0.05. Longitudinal SFI data and thermal withdrawal testing data collected were analysed using two-way repeated-measures ANOVA (group × time) with Sidak's multiple comparisons test. For histological quantification, measurements from multiple sections/fields were first averaged to generate a single specimen-level value per nerve (n = 6 nerves per group) before group-level statistical testing. Unpaired t-test was used for gastrocnemius wet weight and CMAP comparison on the behavioural and electrophysiological studies.

## 3 Results

### 3.1 CES alters stress redistribution and reduces local mechanical gradients on simulated nerve coaptations

Guided by our initial hypothesis that use of an epineurial substitute to support nerve repair can optimize the local mechanical environment, FEA demonstrated that this approach significantly redistributes mechanical loads at the coaptation site. Von Mises stress distributions in the vicinity of the nerve coaptation site were analysed across four repair techniques: single-suture, two-suture, sutureless CES-assisted, and single-suture epineurial substitute-assisted (Fig. 1a-d). In both suture repair models (Fig. 1a and b), the distribution of stress is non-uniform near the coaptation region, with pronounced stress concentrations localized at the suture sites, as indicated by steep stress gradients. Increasing the number of sutures further accentuates these local stress gradients, leading to more abrupt spatial variations in stress around the repair point.

Conversely, both CES-assisted repair models (Fig. 1c and d) exhibit substantially more uniform and less pronounced stress concentrations at the nerve coaptation site. In these cases, elevated stresses are primarily carried by the epineurial substitute, indicating effective load sharing and redistribution of the load away from the nerve-nerve interface. As such, the minimum stress is observed at the repair site itself. Although the addition of a suture in the epineurial substitute-assisted repair (Fig. 1d) introduces a modest increase in non-uniformity of stress load compared with the suture-free epineurial substitute configuration (Fig. 1c), the overall stress magnitude at the coaptation site remains markedly lower than that observed in both suture-only repair models (Fig. 1a and b). The FEA results guided the design of the in vivo animal experimental protocol for epineurial substitute-assisted end-to-end neurorrhaphy with fewer sutures or sutureless as shown in Fig. 1.

### 3.2 CES alters suture configuration and numbers without compromising mechanical stability of nerve coaptation

To validate the mechanical configuration predicted by our FEA models, the biomechanical stability of the coaptation construct was tested immediately after surgery. The uniaxial tensile testing demonstrated that the mechanical competence of the coaptation site was strongly dependent on repair configuration (Fig. 2i and j). Representative load-displacement and stress-strain curves revealed a stepwise increase in load-bearing capacity with increasing suture number, whereas the epineurial substitute-assisted constructs exhibited robust mechanical performance despite reduced or absent transection-site suturing.

Quantitatively, the failure load increased from 0.371 ± 0.078 N in the 1-suture group to 0.545 ± 0.049 N in the 3-suture group, reaching 0.757 ± 0.069 N in the 6-suture group (all *n* = 5). Notably, epineurial substitute reinforcement provided substantial early mechanical strength. Constructs incorporating an epineurial substitute with a single coaptation-site suture achieved a failure load of 0.702 ± 0.034 N, representing an 89% increase compared with the 1-suture group and a 29% increase compared with the 3-suture group. This strength was comparable to that of the 6-suture group (*p* = 0.5331) and significantly greater than that of the 3-suture group (*p* = 0.0022). The sutureless epineurial substitute-assisted construct demonstrated a failure load of 0.647 ± 0.033 N, corresponding to a 75% increase over the 1-suture group and a mechanical strength comparable to that of the 3-suture group (*p* = 0.0635). These findings indicate that epineurial substitute reinforcement can achieve equivalent or superior mechanical stability while substantially reducing the number of coaptation-site sutures required (Fig. 2l).

A similar trend was observed for construct stiffness. The elastic modulus increased from 0.533 ± 0.058 MPa (1 suture) to 0.651 ± 0.070 MPa (3 sutures) and peaked at 1.123 ± 0.124 MPa (6 sutures). Both epineurial substitute-assisted repairs demonstrated comparably elevated modulus values (one transection-site suture = 1.028 ± 0.126 MPa and no sutures = 1.004 ± 0.106 MPa; n = 5), which were not significantly different to the 6-suture neurorrhaphy group (p = 0.5799 and p = 0.3632, respectively) (Fig. 2k).

Intraoperatively, adhesion between the epineurium and the CES was observed to be sufficient to maintain stump alignment even in the absence of suturing (Fig. 2a and b). Under tensile load, conventional suture repairs (1-, 3- and 6-sutures) predominantly failed at the transection site either by rupture of the sutures or epineurial tearing (Fig. 2d–f). Whereas, CES-assisted repairs most often failed at the proximal or distal anchoring sutures, resulting in partial extrusion of one nerve stump from the wrap (Fig. 2g and h). Importantly, even when unilateral displacement occurred, residual epineurial continuity between the proximal and distal nerve stumps was preserved (Fig. 2g). Together this data confirmed the FEM analysis and showed that CES-assisted repair supports a reduction of suture numbers without compromising mechanical stability at the nerve coaptation site.

### 3.3 CES-assisted repair prevents early-stage distal nerve calibre atrophy compared with suturing neurorrhaphy

To further validate if using CES and reduced sutures at the repair site has better nerve repair, we have conducted morphological assessment of the transected nerve. At 4 weeks post-surgery, all suture-only repair groups (Groups A-C) exhibited significantly elevated proximal-to-distal diameter ratios compared with the sham control group (1-suture 1.434 ± 0.367, p = 0.0496; 3-sutures 1.549 ± 0.279, p = 0.0060; 6-suture 1.442 ± 0.309, p = 0.0430; n = 6 per group, Fig. 3a), indicating pronounced distal nerve calibre atrophy. Increasing suture number did not attenuate this effect. In contrast, collagen-assisted repairs (Groups D and E) maintained diameter ratios close to unity (ES+1 S: 1.070 ± 0.071; ES: 1.084 ± 0.082), comparable to sham controls (1.027 ± 0.052; n = 6), reflecting preservation of distal nerve calibre during early recovery (Fig. 3c). Post hoc analysis confirmed significant group differences: the 3-suture group exhibited higher ratios than both CES-assisted groups (p = 0.0135 vs. epineurial substitute with one suture; p = 0.0178 vs. epineurial substitute with sutureless) and sham (p = 0.0060). The 1-suture and 6-suture groups also differed significantly from sham (p = 0.0496 and p = 0.0430, respectively), whereas no significant differences were observed between either CES-assisted group and sham (both ns). By 12 weeks, proximal-to-distal diameter ratios converged across all groups, with no significant differences detected (all ns), indicating progressive restoration of distal nerve calibre irrespective of repair strategy (Fig. 3d).

**Fig. 3:**
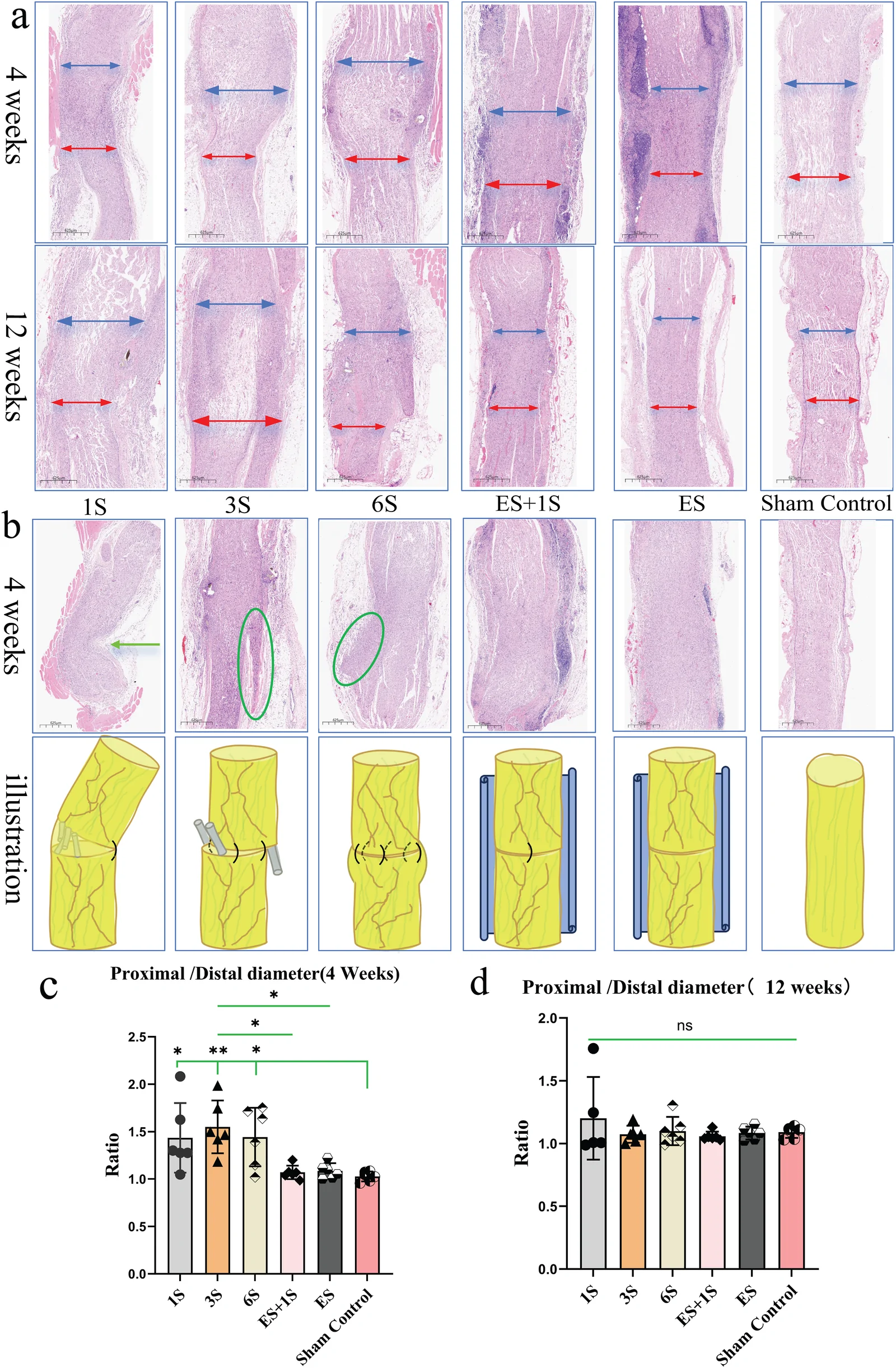
**Morphological characteristics of the nerve coaptation site and proximal-to-distal diameter ratios. (a)** Representative longitudinal views showing the landmarks for diameter measurements at 4 and 12 weeks post-surgery. **(b)** Schematic illustrations and corresponding histological images depicting the morphology of the repair site across experimental groups. **(c, d)** Quantitative analysis of proximal-to-distal diameter ratios at **(c)** 4 weeks (one-way ANOVA (F _(5, 30)_ = 6.004, p = 0.0006; Brown-Forsythe test, p = 0.0717) followed by Tukey's post-hoc test. Exact adjusted p-values are reported. * = p < 0.05, ** = p < 0.01, *** = p < 0.001, ns = not significant.) and **(d)** 12 weeks (one-way ANOVA (F _(5, 29)_ = 0.7182, p = 0.6150; Brown-Forsythe test, p = 0.2286) followed by Tukey's post-hoc test. Exact adjusted p-values are reported. * = p < 0.05, ** = p < 0.01, *** = p < 0.001, ns = not significant.) (Blue and red arrows indicate the proximal and distal nerve stumps, respectively.)

### 3.4 CES-assisted repair mitigates morphological nerve misalignment induced by suturing neurorrhaphy

As shown in Fig. 3b, distinct patterns of fascicular misalignment were observed at 4 weeks among the different repair strategies. In the 1-suture group, three of five nerve segments examined exhibited marked asymmetry at the coaptation interface, characterised by angular deformation of fascicles and lateral displacement of nerve tissue toward one side of the stump, likely reflecting uneven epineurial tension. In the 3-suture group (Group B), most segments displayed a more symmetrical coaptation plane; however, representative sections still demonstrated partial fascicular misalignment, tissue displacement, and focal extrusion beyond the stump margin. In the 6-suture group, increased suture density was associated with reduced lateral displacement at the coaptation site, but excessive tissue approximation frequently resulted in localized bulging of the repaired segment.

In contrast, both CES-assisted repair groups exhibited uniform fascicular alignment across the coaptation region. The nerve stumps demonstrated linear fascicular continuity within the epineurial substitute boundary, without evidence of displacement, enlargement, or extrusion. Overall morphological features in these groups closely resembled those observed in the sham cohort.

### 3.5 CES-assisted repair improves the orientation of cells, myelin and axons at the transection site

To assess early cellular modulation within the repair region, the orientation of nucleated cells was quantified at 4 weeks using the parallel direction ratio (Fig. 4a–d). Myelinated fibre organization and axon regeneration patterns at the transection site were also qualitatively assessed at 4 (Figs.. 4e), 8 and 17 weeks (Fig. S2) post-surgery.

**Fig. 4:**
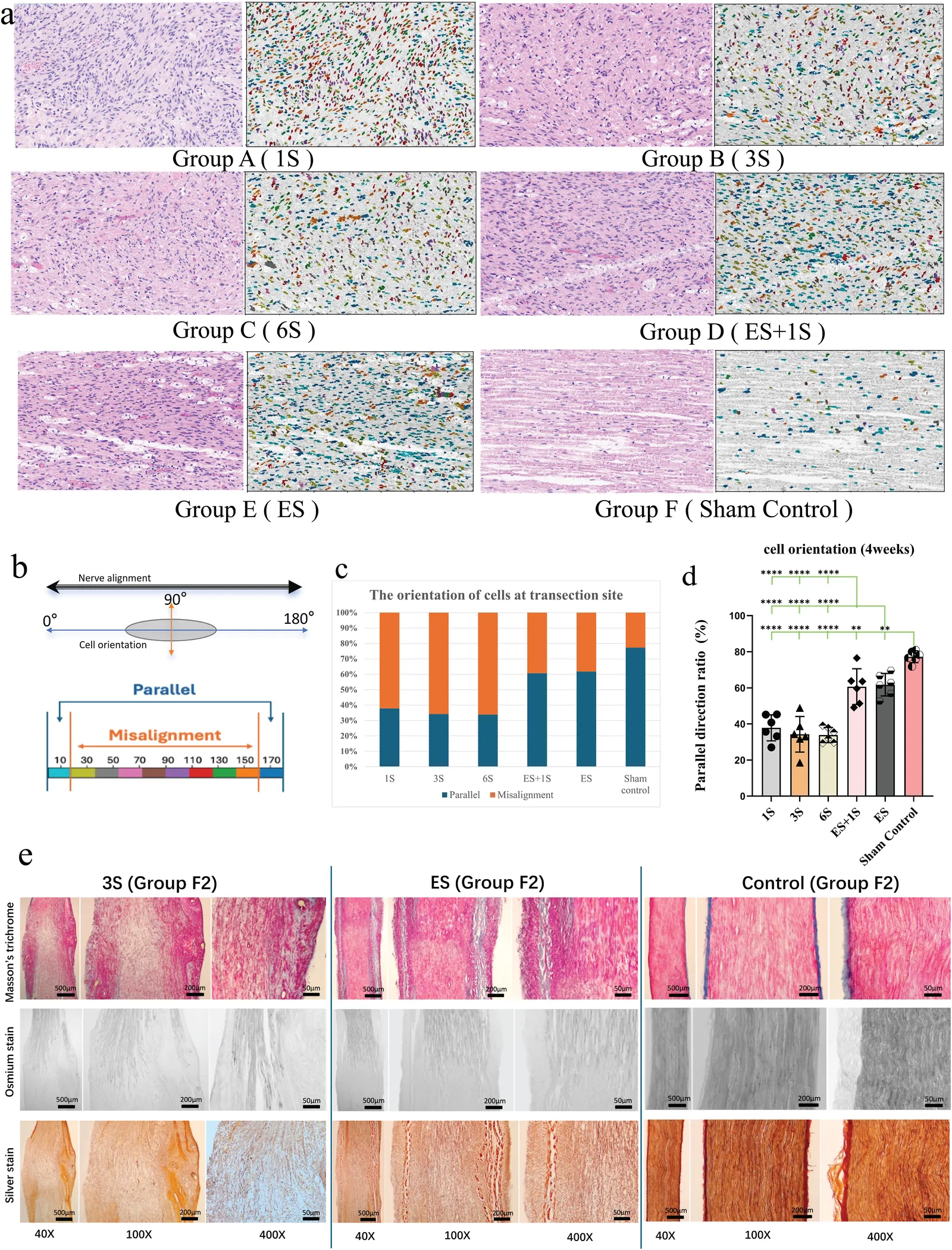
**The orientation of cells at the transection site at 4 weeks. (a)** Representative histological sections (left) and corresponding CellProfiler orientation maps (right) showing the cell orientation patterns at the transection site across all experimental groups. **(b)** Schematic illustration of the orientation quantification workflow, including the longitudinal nerve axis reference and the color-angle mapping approach used to classify cellular orientation as parallel or non-parallel. **(c)** Group-wise orientation distributions depicting the relative proportion of parallel versus non-parallel cells within the transection region. **(d)** Quantitative analysis of parallel direction ratios showing group-level differences in orientation (one-way ANOVA (F _(5, 30)_ = 37.70, p < 0.001; Brown-Forsythe test, p = 0.2993) followed by Tukey's post-hoc test. Exact adjusted p-values are reported. * = p < 0.05, ** = p < 0.01, *** = p < 0.001, **** = p < 0.0001, ns = not significant). (**e**) Representative histological sections at 4 weeks postoperatively from the second animal study, comparing sutureless collagen epineurial substitute-assisted nerve repair and 3-suture repair. Histological evaluation was performed using tissue-specific staining, osmium staining, and Modified Palmgren's silver staining.

Suture-only repair resulted in poor longitudinal cell alignment, with similarly low parallel direction ratios across all suture densities (1-suture: 37.85 ± 7.22%; 3-suture: 34.26 ± 9.86%; 6-suture: 33.83 ± 4.18%; no significant difference).

In contrast, CES-assisted repairs exhibited comparatively better cellular alignment. Both CES-assisted groups showed substantially higher parallel direction ratios (Group D,ES+1; 60.64 ± 9.90% and Group E, ES 61.74 ± 6.21%), representing an increase of approximately 63%–82% over the ratios observed across the suture-only groups (all p < 0.0001). No difference was observed between the two CES-assisted repair strategies (ns, p = 0.9998). As expected, the sham control demonstrated the highest degree of cell orientation (77.25 ± 3.28%), significantly exceeding all experimental repair groups (Fig. 4d).

Based on the equivalent mechanical stability we selected 3-suture and CES+no suture repair configuration to evaluate motor reinnervation, muscle preservation, and neuromuscular recovery for up to 17 weeks. In the CES-no suture group at 4 weeks post-surgery, fragmentation of distal regenerating myelinated axons was observed around and distal to the transection line. The axonal sprouts exhibited a more organized longitudinal orientation, closely paralleling the native nerve axis. In contrast, suture-only repairs demonstrated substantial nerve swelling, disruption of myelinated axon continuity, irregular axonal sprouting, and increased connective tissue deposition within the nerve, all characteristics of Wallerian degeneration following nerve transection (Fig. 4e).

As regeneration progressed, the alignment advantage conferred by CES-assisted sutureless repair was maintained. At both 8 and 17 weeks post-surgery, the morphology and organization of regenerated myelinated axons more closely resembled those of normal nerve tissue compared with the suture-only repair groups, indicating sustained improvement in regenerative architecture over time (Figs. S2 and S3).

### 3.6 CES-assisted repair promotes both epineurial and intraepineurial microvascularization at an early stage post-repair

At 4 weeks post-surgery (Fig. 5a–c), intraepineurial vascularization differed significantly between repair strategies. The 1-suture group demonstrated the lowest vessel area fraction (0.330 ± 0.102%), which was significantly lower than sham surgery. The 3-suture (0.585 ± 0.174%) and 6-suture (0.648 ± 0.155%) groups also had reduced vessel area fractions compared with sham surgery (both p < 0.0001). In contrast, CES-assisted repairs showed markedly greater vascular area fractions (ES+1 S: 1.276 ± 0.228%; ES: 1.592 ± 0.590%), exceeding all suture-only repairs and not significantly different from sham surgery. Notably, the sutureless CES group exhibited a 1.46- to 2.82-fold increase in vascular area compared to the suture-only groups. Although the sutureless CES repair exhibited the highest mean values, there was no significant difference between the two epineurial substitute-assisted repairs. A similar pattern was observed for intraepineurial vessel density. All suture-only repairs displayed comparably low densities (42.61-53.94 n/mm^2^), with no significant differences between 1-, 3- and 6-suture groups. By contrast, both CES-assisted repairs showed substantially higher vessel densities (∼120 n/mm^2^), significantly exceeding all suture-only groups, and comparable to sham surgery values.

**Fig. 5:**
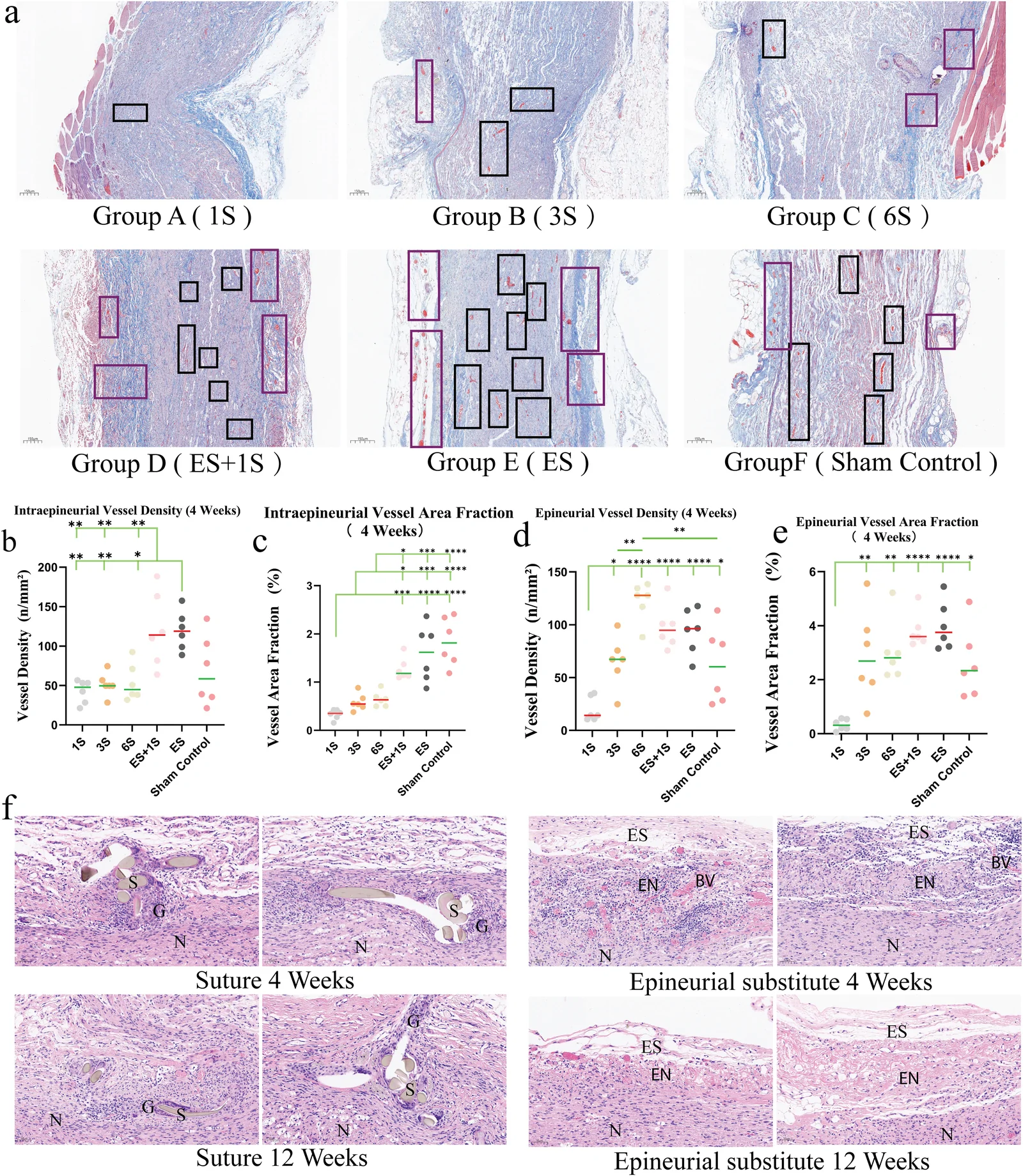
**Microvascular remodelling and inflammatory features at the nerve coaptation site. (a)** Representative Masson's trichrome-stained sections at 4 weeks, illustrating epineurial and intraepineurial microvessels across repair groups. **(b, c)** Quantitative analysis of intraepineurial vessel density **(b)** (one-way ANOVA (F _(5, 30)_ = 18.72, p < 0.0001; Brown-Forsythe test, p < 0.0001) followed by Tukey's post-hoc test. Exact adjusted p-values are reported. * = p < 0.05, ** = p < 0.01, ns = not significant) and vessel area fraction **(c)** (one-way ANOVA (F _(5, 30)_ = 7.629, p = 0.0001; Brown-Forsythe test, p = 0.0441) followed by Tukey's post-hoc test. Exact adjusted p-values are reported. * = p < 0.05, ** = p < 0.01, *** = p < 0.001, **** = p < 0.0001, ns = not significant) at 4 weeks. **(d, e)** Quantification of epineurial vessel density **(d)** (one-way ANOVA (F _(5, 30)_ = 14.02, p < 0.0001; Brown-Forsythe test, p = 0.0969) followed by Tukey's post-hoc test. Exact adjusted p-values are reported. * = p < 0.05, ** = p < 0.01, *** = p < 0.001, **** = p < 0.0001, ns = not significant) and vessel area fraction **(e)** (one-way ANOVA (F _(5, 30)_ = 8.798, p < 0.0001; Brown-Forsythe test, p = 0.1086) followed by Tukey's post-hoc test. Exact adjusted p-values are reported. * = p < 0.05, ** = p < 0.01, *** = p < 0.001, **** = p < 0.0001, ns = not significant) at 4 weeks. **(f)** Representative H&E-stained sections at 4 and 12 weeks, showing cellular infiltration and matrix remodelling within the suture tracks and the epineurial substitute-assisted repair zones. (ES: Epineurial Substitute; N: Nervous Tissue; EN: Epineurial substitute-Nerve Interface; BV: Blood Vessel; S: Suture; G: Giant Cell. N = 6 per group.)

In the epineurial compartment (Fig. 5a–d,e), vascularization also varied markedly among groups. The 1-suture group exhibited the lowest vessel density (19.80 ± 11.56 n/mm^2^), significantly lower than all other repair strategies and sham surgery (p = 0.0404). Vessel density increased with suture number, peaking in the 6-suture group (122.30 ± 18.18 n/mm^2^), which exceeded both the 3-suture group and sham surgery. The CES-assisted repairs demonstrated intermediate-to-high vessel densities comparable to the 6-suture and sham surgery groups (ES+1 S vs. Sham Control: p = 0.1232; ES vs. Sham Control: p = 0.2098), and no difference between the two CES-assisted repairs. Consistent trends were observed for epineurial vessel area fraction. The 1-suture group showed minimal vascular occupancy (0.329 ± 0.208%), significantly lower than all other groups, whereas no significant differences were detected among the 3-suture, 6-suture, CES-assisted, and sham surgery groups, all of which exhibited similarly elevated vascular area fractions.

### 3.7 Suture induces prolonged local inflammatory responses at the coaptation site

Representative H&E-stained sections illustrating the repair architecture and tissue interface are shown in Fig. 5f. At 4 weeks post-surgery, prominent inflammatory cell infiltration was observed along the suture tracts. The response was characterised by a pronounced foreign-body reaction, with dense aggregates of multinucleated giant cells and lymphocytes forming well-demarcated inflammatory foci around residual suture material. This inflammatory response persisted at 12 weeks and 17 weeks post-surgery (Fig. S2), and was accompanied by the development of a prominent fibrotic capsule encasing the sutures, resulting in ongoing structural distortion at the repair interface.

In contrast, the inflammatory response at the repair interface of CES-assisted repair 4 weeks post-surgery was dominated by mild lymphocytic infiltration, with no evidence of a foreign-body giant cell reaction. This response appeared transient, and was largely resolved by 12 weeks and had fully subsided by 17 weeks post-surgery (Fig. S2), coinciding with the emergence of a more uniform epineurial-like structure. Concomitantly, the CES-assisted repair zone demonstrated progressive morphological remodelling, with improved nerve fibre continuity and a more regular longitudinal organization across the transection interface. These changes were associated with a marked reduction in structural heterogeneity, indicating more favorable tissue integration and maturation over time.

A gradual process of the epineurial substitute degradation, accompanied by epineurial remodelling, was observed across all four time points, while the integrity of the intraneural structures was maintained throughout the observation period. At 4 weeks post-surgery, the epineurial substitute had begun to resorb, although its overall linear continuity remained intact. This stage was associated with peak lymphocyte infiltration. At 8 weeks, further degradation of the collagen fibres was evident, accompanied by tissue ingrowth and a reduction in inflammatory cell infiltration. At 12 weeks, scattered residual collagen fibres were still detectable within the regenerated epineurium, while the inflammatory response had largely resolved. By 17 weeks, the collagen material had completely resorbed and was replaced by regenerated epineurial tissue, with no evidence of persistent inflammation.

### 3.8 CES-assisted repair improves early sciatic functional recovery, facilitates earlier sensory recovery, and enhances final motor function

Footprint analysis demonstrated clear, time-dependent motor recovery following sciatic nerve repair (Fig. 6a–f). All experimental groups exhibited an immediate postoperative decline in Sciatic Functional Index (SFI), followed by gradual improvement over 12 weeks, although full recovery to sham surgery levels was not observed in any of the experimental groups.

**Fig. 6:**
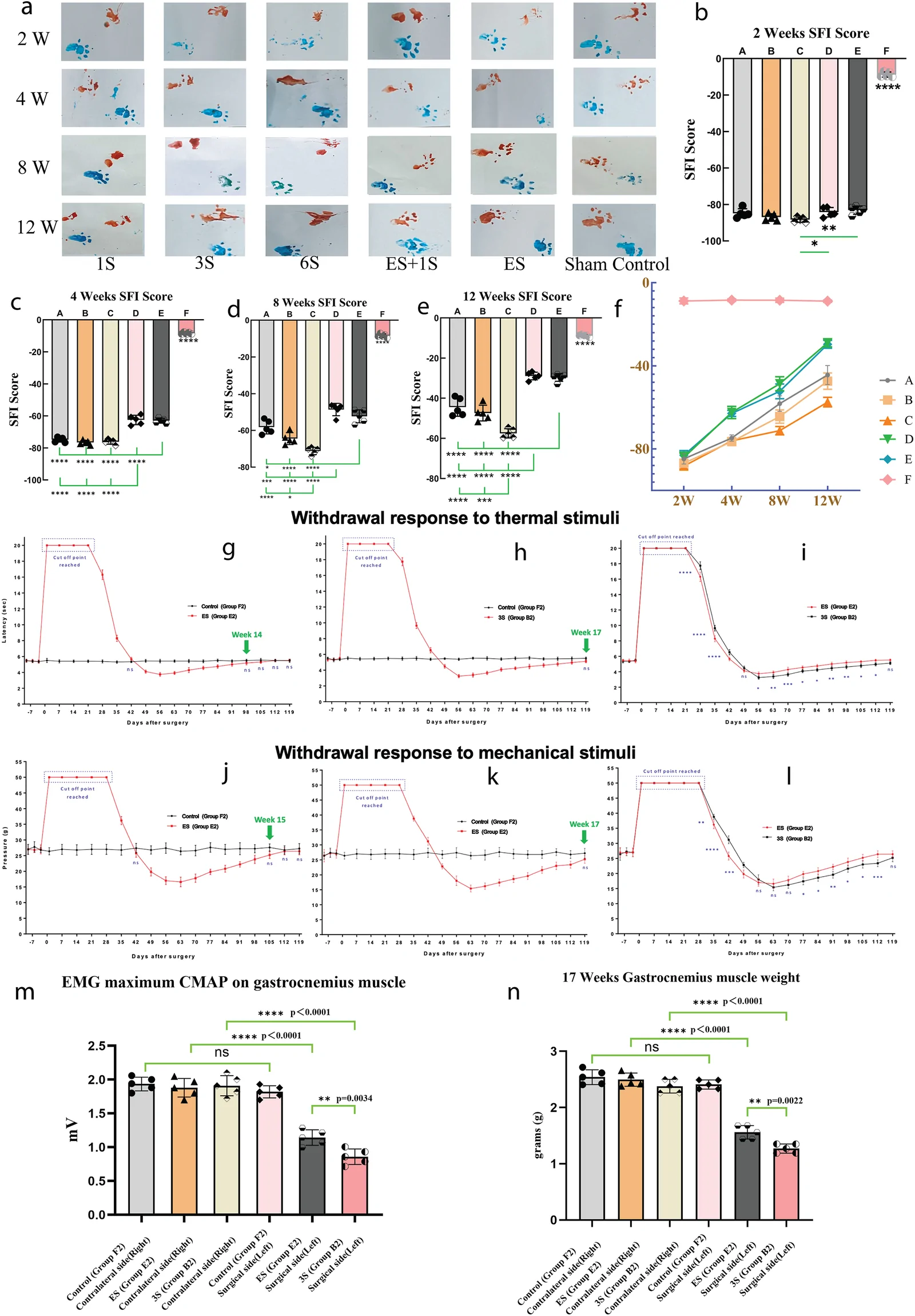
**Functional recovery and muscle atrophy analysis post-nerve repair. (a)** Representative walking track footprints at 2, 4, 8, and 12 weeks, illustrating gait progression across groups. **(b-e)** Quantitative assessment of the Sciatic Functional Index (SFI) at 2 **(b)**, 4 **(c)**, 8 **(d)**, and 12 **(e)** weeks postoperatively. **(f)** Temporal SFI recovery curves depicting the kinetics of functional improvement across all repair conditions. **(g-i)** Mechanical withdrawal testing for assessment of sensory recovery (2way ANOVA, 2way ANOVA, * = p ≤ 0.05, ** = p ≤ 0.01, *** = p ≤ 0.001, **** = p ≤ 0.0001, ns = not significant, n = 5, mean ± SD) **(g)** Thermal withdrawal testing of sutureless repair with collagen epineurial substitute (Group E2) versus the control nerves; showing transient hypoesthesia followed by hyperaesthesia and recovery to baseline by week 14. **(h)** Thermal withdrawal testing of 3x suture (Group B2) versus the control healthy nerves showing transient hypoesthesia followed by hyperaesthesia and recovery to baseline by week 17. (**i**) Thermal withdrawal testing comparison of Group E2 and Group B2; Group E2 demonstrated faster sensory recovery, reduced hyperaesthesia, and earlier return to baseline compared with Group B2. **(j-l)** Mechanical withdrawal testing for assessment of sensory recovery (2way ANOVA, 2way ANOVA, * = p ≤ 0.05, ** = p ≤ 0.01, *** = p ≤ 0.001, **** = p ≤ 0.0001, ns = not significant, n = 5, mean ± SD), (**j)** Thermal withdrawal testing of Group E2 versus the control nerves; showing transient hypoesthesia followed by hyperaesthesia and recovery to baseline by week 15. **(k)** Mechanical withdrawal testing Group B2 versus the control healthy nerves showing transient hypoesthesia followed by hyperaesthesia and recovery to baseline by week 17. (**l**) Mechanical withdrawal testing comparison of Group E2 and Group B2; Group E2 show significantly better recovery from initial anesthesia, reach less hyperaesthesia and show faster recovery from hyperaesthesia.**(m)** Maximum compound muscle action potential on gastrocnemius muscles that was produced in Group B2, Group E2, Group F2 at the end of experiment (17 weeks post-surgery); contralateral muscles in each group were used as controls. **(n)** Weight of gastrocnemius muscles measured in Group B2, Group E2, Group F2 at the end of experiment (17 weeks post-surgery); contralateral muscle in each group served as a control.

At 2 weeks post-surgery, all repair groups exhibited severe functional impairment with similarly low SFI values, whereas sham-operated animals maintained near-normal gait function (Fig. 6b). By 4 weeks, functional outcomes diverged between repair strategies, with both CES-assisted groups demonstrating significantly improved SFI values compared with all suture-only repair groups (Fig. 6c).

This difference was maintained throughout the study period. At both 8 and 12 weeks, the epineurial substitute-assisted groups continued to demonstrate superior functional recovery compared with conventional neurorrhaphy groups (Fig. 6d and e). Among the suture-only repairs, the 6-suture group consistently exhibited the poorest functional outcome, whereas no significant difference was observed between the 1-suture and 3-suture groups. By 12 weeks, SFI values in the CES-assisted groups approached −30, indicating substantially improved functional recovery relative to all suture-only repair groups (Fig. 6e).

The SFI was used as a screening tool to assess overall functional recovery following nerve repair. To provide a more detailed assessment of sensory and motor recovery based on the equivalent mechanical stability between the epineurial substitute-assisted sutureless repair (ES Group E2) and the conventional 3-suture repair (Group B2), behavioural (withdrawal threshold tests) and compound muscle action potential (CMAP) testing was conducted weekly over 17 weeks post-surgery. Both groups exhibited an initial period of sensory loss following surgery, characterised by prolonged thermal and mechanical withdrawal latencies. Sensory function began to recover by 4 weeks post-surgery and returned to baseline levels by approximately week 6. Thereafter, both groups developed transient hypersensitivity, which peaked around week 8 post-surgery. The hypersensitivity gradually resolved over time, with sensory responses returning to baseline by week 14 for thermal withdrawal and week 15 for mechanical withdrawal (Fig. 6g,j) in the ES group (E2), compared with week 17 for both thermal and mechanical withdrawal (Fig. 6h,k) in 3-suture group (B2).

Compared with conventional suture repair, epineurial substitute-assisted sutureless repair demonstrated significantly faster, approximately 2-3 weeks, recovery from postoperative sensory loss, reduced severity of hyperaesthesia, and an earlier return to baseline sensory function than conventional neurorrhaphy (Fig. 6i and l). However, despite the differences in the rate of recovery, both repair strategies achieved comparable sensory outcomes by 17 weeks post-surgery.

Both ES (E2) and 3-suture (B2) groups demonstrated significantly reduced gastrocnemius CMAP amplitudes and gastrocnemius muscle weights compared with their respective contralateral controls (Fig. 6m and n). CMAP amplitude is proportional to the number of regenerated motor axons and the size of the corresponding motor units (i.e., the muscle fibres innervated by a single motor axon) within the target muscle [27]. These findings indicate that complete restoration of motor innervation and muscle function had not been achieved by 17 weeks post-surgery.

Despite the differences observed in motor functional recovery as assessed by the SFI, gross muscle mass measurements did not demonstrate corresponding differences during the early stages of regeneration. No significant differences in gastrocnemius muscle atrophy were detected between treatment groups at either 4 or 12 weeks post-surgery (Figs. S4 and S5). However, following the plateau of motor functional recovery, the CES group (E2) exhibited significantly greater gastrocnemius CMAP amplitudes and muscle weights than the 3-suture group (B2; Fig. 6m and n). These findings suggest that epineurial substitute-assisted sutureless repair promotes superior motor axon regeneration, functional reinnervation, and preservation of target muscle mass compared with conventional suture repair.

## 4 Discussion

The integration of computational modelling and biomechanical validation in this study reveals the potential for a shift in the use of sutures in peripheral nerve repair. Using an epineurial substitute wrap to augment surgical repair of peripheral nerves, it is feasible to transfer point-load fixation at the repair site to a distributed-load stabilization away from the repair site, as well as using fewer sutures to stabilize the coaptation in end-to-end neurorrhaphy.

FEA demonstrated that mechanical stress was highly concentrated at suture sites in conventional neurorrhaphy, whereas a CES-assisted repair strategy resulted in a more homogeneous stress distribution. Importantly, this redistribution of stress did not compromise the mechanical stability of the repair. Mechanical testing showed that, compared with conventional neurorrhaphy, epineurial substitute-assisted repair achieved non-inferior stability of the coaptation, despite the use of fewer or no sutures at the transection site. Beyond preserving mechanical integrity, the epineurial substitute-assisted approach provided handling characteristics comparable to traditional suturing, reduced direct manipulation of the injured nerve, facilitated intraoperative verification of fascicular alignment, and minimised secondary iatrogenic sharp neural injury during repair [28].

The predicted dynamics of stress distribution are echoed at both the tissue and cellular levels. At the tissue level, angular deformation of nerve fascicles caused by uneven epineurial traction from single-stitch neurorrhaphy can be mitigated by epineurial substitute assistance (ES+1s, Fig. 3b), indicating a more uniform distribution of stress along the nerve. At the cellular level, quantitative analysis of the cell orientation at the transection site revealed significantly better alignment in the epineurial substitute-assisted group. It has been previously shown that even minimal local mechanical stress can influence cellular orientation [29]. By creating a tension-free local microenvironment, the epineurial substitute-assisted group guided more cells, myelination and axonal regeneration along the longitudinal axis of the nerve. In contrast, the use of sutures created stress concentration in the critical regeneration zone, which disrupted cell and axonal alignment. Thus, the epineurial substitute approach more closely recapitulates the native cellular orientation observed in healthy peripheral nerves. We acknowledge that the current assessment of parallel cellular orientation was based primarily on H&E-stained sections and morphological evaluation, which cannot definitively confirm the identity of the aligned cells as Schwann cells. Nevertheless, overall cellular alignment remains a biologically meaningful indicator of the regenerative microenvironment. During peripheral nerve regeneration, Schwann cells organize into longitudinally aligned Bands of Büngner [27,30] that provide essential guidance pathways for regenerating axons, while fibroblasts contribute to the establishment and maintenance of this regenerative architecture through extracellular matrix remodelling and Schwann cell–fibroblast interactions. Therefore, enhanced cellular anisotropy is likely to reflect a microenvironment more conducive to axonal regeneration, even in the absence of definitive cell-type identification. This interpretation is further supported by the silver-stained histology findings, which demonstrated improved axonal organization and regeneration in the epineurial substitute-assisted group.

Re-establishing the vasa nervorum, the vascular network of peripheral nerves, is critical for successful nerve regeneration [31]. In the present study, increasing the number of sutures to improve nerve stump approximation appeared to promote microvascularization predominantly within the epineurium. However, epineurial substitute-assisted repair resulted in a more pronounced enhancement of intra-epineurial vascularization, including endoneurial and perineurial vascularization. Given that revascularization and intraneural blood flow at the nerve repair site are highly sensitive to, and negatively affected by, mechanical tension [32,33], the tension-free microenvironment at the coaptation site created by epineurial substitute assistance likely contributed to the observed vascular improvement.

In parallel, compared with conventional neurorrhaphy in which the repair site is directly exposed to surrounding tissue fluids, the semi-permeable CES used in this study may help maintain a transient and controlled hypoxic microenvironment during the early phase of regeneration. This environment may facilitate early intraneural revascularization, potentially through hypoxia-inducible factor-1α (HIF-1α) and vascular endothelial growth factor (VEGF)-mediated signaling pathways [34,35]. As the CES gradually degrades and microcirculation is restored, the transient hypoxic state would be expected to diminish, while the tension-free coaptation environment is preserved throughout the regenerative process.

The collagen epineurial substitute acts as a physical barrier to connective tissue infiltration, thereby protecting newly formed repair tissue from invasion by perineural scar tissue [[36], [37], [38], [39], [40]], which is also beneficial for preserving intra-epineurial vascularization. In the epineurial region, the collagen epineurial substitute provides an additional extracellular matrix scaffold that facilitates metabolic exchange and diffusion of nutrients and growth-promoting factors, thereby further enhancing epineurial vascularization [41,42].

Early restoration of the vasa nervorum is likely a key mechanism underlying the prevention of distal fascicular atrophy, as well as the enhanced early motor recovery and accelerated sensory restoration observed in the collagen epineurial substitute-assisted repair group. Interestingly, despite significant improvements in SFI, no differences in gastrocnemius muscle outcomes were detected between groups during the early stages of regeneration. Previous studies using comparable rat sciatic nerve repair models have demonstrated that SFI typically undergoes a rapid phase of recovery within the first 8–12 weeks following injury, after which further improvement occurs at a substantially slower rate [43]. Consistent with this pattern, our study demonstrated significantly greater gastrocnemius muscle weight and CMAP amplitude in the collagen epineurial substitute-assisted repair group at 17 weeks postoperatively. These findings suggest that the early regenerative advantages conferred by improved vascularization and a favorable repair microenvironment may ultimately translate into superior long-term motor reinnervation and muscle preservation, even after SFI recovery has largely plateaued.

Beyond redistribution of mechanical stress, epineurial substitute-assisted repair in this study demonstrated additional advantages over conventional neurorrhaphy by attenuating long-term adverse inflammation and preventing fascicular malalignment. In clinical practice, 9-0 or 10-0 non-absorbable nylon sutures are routinely used for epineurial neurorrhaphy. Consistent with previous reports [44,45], use of sutures resulted in a pronounced foreign body response at early time points, characterised by multinucleated giant cells that persisted into later stages of repair. This was accompanied by granulation tissue formation and progressive collagen deposition, indicative of a sustained, suture-driven inflammatory and fibrotic microenvironment. In contrast, epineurial sutures confined to the proximal and distal epineurial substitute-nerve junctions, spatially separated from the critical zone of axonal regeneration, did not appear to elicit the same inflammatory response. The epineurial substitute itself demonstrated rapid tissue integration followed by gradual resorption, which was associated with a transient local inflammatory response that subsided over time.

Further, the epineurial substitute enclosed all the fascicles from the transected nerve ends, preventing axonal escape from insufficient sealing of the coaptation. When nerve fascicles are left unsupported, exposed, or subjected to abnormal mechanical stress following injury or surgical intervention, they are prone to disorganised regeneration, neuroma formation, and aberrant axonal sprouting, facilitating the transition from acute nerve injury to chronic neuropathic pain [46,47]. Our histological findings suggest that use of wraps to prevent axonal escape may contribute to reduced incidence of neuroma-related pain, however, this remains to be directly tested [48].

The primary objective of this study was to optimize the coaptation technique rather than merely evaluating the superiority of biomaterials. Despite the proven advantages of various scaffolds [49,50], many clinical neurosurgeons still rely on their intraoperative tactile feedback and prefer three to four epineural sutures to balance nerve alignment and mechanical stability. Our study proposes a shift in this paradigm by demonstrating, using FEA-guided design, the benefits of reducing the number of sutures at the transection site and relocating fixation to the proximal and distal ends through epineurial substitute assistance. Use of the epineurial substitute additionally allows surgeons to assess coaptation quality immediately while minimizing suture-induced adverse effects. In clinical practice, using a single epineural stitch to ensure proper nerve-end approximation before applying the collagen membrane represents a more translatable and widely acceptable strategy, respecting traditional surgical intuition while leveraging the benefits of modern biomaterial design.

Previous in vivo evidence has indicated that nerve repairs relying solely on fibrin glue are often mechanically insufficient to withstand physiological stretching, frequently resulting in complete detachment or significant elongation of the nerve stumps within the first four weeks post-operation [51]. Our findings address this critical vulnerability by demonstrating that epineurial substitute functions as a load-sharing scaffold. By redistributing mechanical stress away from the interface, this approach provides the necessary structural reinforcement that simple sealants lack, thereby preventing the detachment issues reported in earlier sutureless models.

Despite these advantages, several limitations of the present study should be acknowledged. First, the parallel direction ratio was derived from H&E-based morphological analysis and therefore reflects overall cellular orientation rather than the alignment of a specific cell population. Without immunohistochemical characterization, we were unable to distinguish Schwann cells from fibroblasts, inflammatory cells, or other cell types within the regenerating tissue. Consequently, the observed cellular alignment along the nerve can only be interpreted as an indicator of a more organized landscape of nerve repair rather than direct evidence of Schwann cell alignment. Future studies incorporating cell-specific immunohistochemical markers, including S100, SOX10, MBP, and NF200, will be necessary to validate the cellular composition and organization of the regenerating nerve. Second, biological and mechanical outcomes may vary with different conduit materials, including synthetic polymers such as PLGA, PGA, and PCL, as well as natural materials like chitosan [52], due to differences in stiffness and degradation kinetics. Third, postoperative factors relevant to clinical practice, such as immobilization and rehabilitation, play important roles in functional recovery but are difficult to standardize in animal models. Finally, the finite element analysis employed an idealized longitudinal model based on assumptions of linear elasticity and isotropic material behaviour, which may not fully capture the viscoelastic, anisotropic, and multi-axial mechanical characteristics of peripheral nerves in vivo. These simplifications may have influenced the absolute magnitude and spatial distribution of the predicted stresses and strains. Nevertheless, the model was intended primarily for comparative evaluation of different repair configurations under standardized conditions. Future studies incorporating more physiologically representative constitutive models, sensitivity analyses, and in situ mechanical measurements may further improve biomechanical prediction and clinical translatability.

## 5 Conclusion

Collagen epineurial substitute provides mechanical and biological advantages to deploying sutures away from the coaptation site, thereby preserving nerve fascicular architecture, promoting early pro-regenerative vascularization, and minimizing suture-related trauma for early functional recovery.

## Data availability

Data generated or analysed during this study are included in this published article and its supplementary information files. Raw data that support the findings of this study are available from the corresponding author (R.R and M.H.Z) upon reasonable request.

## Authorship contributions

Yongquan Wang: Investigation; Formal analysis; Data curation; Writing - original draft; Validation; Methodology.

Zoran Pletikosa: Investigation; Validation; Data curation; Methodology.

Peilin Chen: Visualization; Software; Data curation.

Jiayue Li: Visualization; Software; Data curation.

Ziming Chen: Visualization.

Cameron Bright: Methodology.

Sharon Chu: Conceptualization.

Alex O'Beirne: Conceptualization.

David Brogan: Conceptualization.

Brendan F. Kennedy: Resources.

Harry Hoyen: Conceptualization; Writing - review & editing.

Haotian Li: Methodology.

Daohui Li: Methodology.

Clair Lee: Conceptualization; Writing - review & editing.

Lingqiang Chen: Funding acquisition; Resources; Supervision.

Rui Ruan: Project administration; Supervision; Validation; Formal analysis; Data curation; Writing - original draft; Writing - review & editing.

Minghao Zheng: Conceptualization; Funding acquisition; Project administration; Supervision; Writing - review & editing.

## Ethics declaration

This study was conducted in accordance with the ARRIVE (Animal Research: Reporting of In Vivo Experiments) guidelines. This study was approved by the Kunming Medical University. (Approval No. #KMMU20231515) And Western Sydney University (ACEC: A10622).

## Declaration of generative AI in scientific writing

AI tool, Chat GPT was used to assist in the creation of illustrations for the graphical abstract and to identify typographical errors in the manuscript. The authors reviewed and edited all AI-generated content as necessary and take full responsibility for the accuracy, integrity, and content of the published article.

## Funding statement

The animal study of this work was supported by Key Program of Associated Project of Yunnan Province Science & Technology Department and 10.13039/501100003996Kunming Medical University Basic Research for Application (Grant No.: 202501AY070001-013), Yunnan Revitalization Talent Support Program, 535 Talent Project of First Affiliated Hospital of 10.13039/501100003996Kunming Medical University (Grant No.: 2023535D05), and Yunnan Health Training Project of High-Level Talent. (Grant No. L-2024014).

## Declaration of competing interest

Minghao Zheng is inventor on intellectual property related to the device used in this study. The associated technology has been licensed to Orthocell Ltd, Australia, in which Minghao Zheng holds shares/equity interests. Clair Lee receives salary support from Orthocell. All other authors declare no competing financial or non-financial interests.
