# Supplementary material for: Collagen epineurial substitute-assisted neurorrhaphy is superior to conventional end-to-end neurorrhaphy in a rat model of peripheral nerve injury
Source: J Orthop Translat. 2026 Sep 21;60:101197. doi: 10.1016/j.jot.2026.101197 (PMC13631819; doi:10.1016/j.jot.2026.101197)
Supplement: Supplementary file 1 [file mmc1.doc]

**SUPPLEMENTARY DATA**

Further contextual details that could not be accommodated in the manuscript are specified below.

Figure S1. Experimental design and study flowchart.

Figure S2. Representative histological sections at 8 weeks postoperatively from the second animal study, comparing sutureless collagen epineurial substitute-assisted nerve repair and 3-suture repair. Histological evaluation was performed using tissue-specific staining, osmium staining, and Modified Palmgren’s silver staining.

Figure S3. Representative histological sections at 17 weeks postoperatively from the second animal study, comparing sutureless collagen epineurial substitute-assisted nerve repair and 3-suture repair. Histological evaluation was performed using tissue-specific staining, osmium staining, and Modified Palmgren’s silver staining.

Figure S4 Wet weight ratio of gastrocnemius muscles ( Surgery site/contralateral side) measured in 4 weeks for Part 1 animal study

Figure S5 Wet weight ratio of gastrocnemius muscles ( Surgery site/contralateral side) measured in 12 weeks for Part 1 animal study

**Captions for supplementary movies**

**Supplementary movie 1**

Intraoperative tension assessment in epineurial substitute-assisted repair.

**Supplementary movie 2**

Mechanical testing of the repair site using sutures

**Supplementary movie 3**

Mechanical testing of epineurial substitute-assisted nerve repairs

**Supplementary movie 4**

Representative footprints during the acute denervation phase (2 weeks post-surgery), exhibiting a collapsed toe pattern and indistinct intermediary toe spread.

**Supplementary movie 5**

Morphological restoration of footprints at the 12-week endpoint. Note the transition from a collapsed toe pattern to a normalized digital gait.
